# HIV Envelope Trimer Specific Immune Response Is Influenced by Different Adjuvant Formulations and Heterologous Prime-Boost

**DOI:** 10.1371/journal.pone.0145637

**Published:** 2016-01-04

**Authors:** Juliana de Souza Apostólico, Silvia Beatriz Boscardin, Márcio Massao Yamamoto, Jethe Nunes de Oliveira-Filho, Jorge Kalil, Edecio Cunha-Neto, Daniela Santoro Rosa

**Affiliations:** 1 Department of Microbiology, Immunology and Parasitology, Federal University of São Paulo (UNIFESP/EPM), São Paulo, Brazil; 2 Department of Parasitology, Institute of Biomedical Sciences, University of São Paulo, São Paulo, Brazil; 3 Heart Institute (InCor), University of São Paulo—School of Medicine, São Paulo, Brazil; 4 Institute for Investigation in Immunology—INCT, São Paulo, Brazil; 5 Laboratory of Clinical Immunology and Allergy—LIM60, University of São Paulo- School of Medicine, São Paulo, Brazil; Commissariat a l'Energie Atomique(cea), FRANCE

## Abstract

The development of a preventive vaccine against human immunodeficiency virus (HIV-1) infection is the most efficient method to control the epidemic. The ultimate goal is to develop a vaccine able to induce specific neutralizing, non-neutralizing antibodies and cellular mediated immunity (CMI). Humoral and CMI responses can be directed to glycoproteins that are normally presented as a trimeric spike on the virus surface (gp140). Despite safer, subunit vaccines are normally less immunogenic/effective and need to be delivered together with an adjuvant. The choice of a suitable adjuvant can induce effective humoral and CMI that utterly lead to full protection against disease. In this report, we established a hierarchy of adjuvant potency on humoral and CMI when admixed with the recombinant HIV gp140 trimer. We show that vaccination with gp140 in the presence of different adjuvants can induce high-affinity antibodies, follicular helper T cells and germinal center B cells. The data show that poly (I:C) is the most potent adjuvant to induce specific CMI responses evidenced by IFN-γ production and CD4^+^/CD8^+^ T cell proliferation. Furthermore, we demonstrate that combining some adjuvants like MPL plus Alum and MPL plus MDP exert additive effects that impact on the magnitude and quality of humoral responses while mixing MDP with poly (I:C) or with R848 had no impact on total IgG titers but highly impact IgG subclass. In addition, heterologous DNA prime- protein boost yielded higher IgG titers when compare to DNA alone and improved the quality of humoral response when compare to protein immunization as evidenced by IgG1/IgG2a ratio. The results presented in this paper highlight the importance of selecting the correct adjuvant-antigen combination to potentiate desired cells for optimal stimulation.

## Introduction

HIV-1 infection and the incurable disease it causes, acquired immunodeficiency syndrome (AIDS), are still major global health problems. Since the development of antiretroviral drugs, millions of HIV-infected individuals have been saved from progression to AIDS. Although a lot of progress was accomplished in prevention strategies, including pre-exposure prophylaxis [1][2][3], the ultimate control of the epidemic will mostly rely on a preventive vaccine. The major challenge for the development of such vaccine resides in finding correlates of protection that need to be elicited during vaccination.

For example, studies in a small fraction of HIV infected individuals that do not progress to AIDS have shown that protection can be mediated by broadly neutralizing antibodies (bNabs) [4][5][6]. In addition, non-neutralizing antibodies might also have the potential to afford partial protection against HIV-1 infection [7] through antibody-dependent cell-mediated cytotoxicity (ADCC) and antibody-dependent cell-mediated viral inhibition (ADCVI) [8][9]. On the other hand, cellular immune responses, including CD8^+^ T lymphocytes [10], NK cells [11], and CD4^+^ T lymphocytes [12][13], can also mediate control of viremia in HIV-1-infected individuals.

In HIV-1 infected patients, neutralizing (Nabs) and non-neutralizing antibodies are mainly directed against the glycoproteins from the virus envelope (env). The precursor of the envelope protein, gp160, forms a trimer (gp120/gp41)_3_ that is cleaved by a furin-like protease into two non-covalently associated fragments: gp120 for receptor binding and gp41 for membrane fusion.

Unfortunately, vaccination approaches using recombinant HIV envelope proteins and derivatives specifically engineered to elicit bNabs have all been disappointing to date. Two phase III clinical trials of a prophylactic HIV vaccine candidate (VAX003 and VAX004) using the monomeric version of the envelope glycoprotein subunit (gp120) in alum adjuvant were undertaken. The results of these trials demonstrated that the antibodies elicited by monomeric gp120 failed to prevent infection, neutralize HIV primary isolates, reduce viral loads or delay disease progression [14][15][16]. These disappointing results may be explained by the fact that a monomeric version of gp120 was used, while env glycoproteins are normally presented as a trimeric spike (gp120/gp41)_3_ on the virus surface. Most recently, the RV144 HIV-1 vaccine trial was the first to demonstrate some evidence of protection against HIV-1 infection in the absence of serum Nabs, with an estimated vaccine efficacy of 31.2% [17]. This vaccine regimen consisted of four doses of recombinant canarypox priming (vCP1521) followed by two doses of the recombinant HIV-1 gp120 protein (AIDSVAX). Comparison of the immune responses in the vaccinees and placebo groups revealed that it is possible that non-neutralizing antibodies and CMI reduced the infection rate in RV144 [18][19][20][21][22][23][24]. In addition, a vaccination study in macaques showed protection from infection in the absence of Nabs, suggesting that non-neutralizing might indeed protect [25].

Few antibodies raised by gp120 monomers effectively bind assembled HIV-1 envelope glycoprotein trimers [26]. Also, Nabs generally bind with higher affinity to membrane-associated trimeric forms of env when compared to monomeric forms of gp120 [27]. Therefore, different strategies have been developed to generate soluble, stable, recombinant trimers as antigens that resemble the native env protein and better stimulate specific immune responses. Different groups [28][29][30][31] reported several modifications to stabilize gp140 trimers, like disruption of the proteolytic cleavage site between gp120 and gp41, introduction of cysteines that form intersubunit disulfide bonds and addition of trimerization motifs. Studies evaluating the properties of trimeric gp140 demonstrated that it bound to soluble CD4 and CCR5, retained its antigenicity and was able to induce/ bind to Nabs more efficiently than monomeric gp120 [32, 33].

The development of a new generation of potent vaccines for preventing disease caused by infectious pathogens relies on the selection and usage of suitable adjuvants [34]. Their use is essential to improve humoral and CMI to the vaccine antigens leading ultimately to long-lasting immunity and protection. Many diverse classes of compounds have been assessed as adjuvants, including mineral salts, microbial products, emulsions, saponins, cytokines, polymers, microparticles, and liposomes [35]. Adjuvants can lead to a reduction of the dose and/or number of immunizations for a given vaccine [36] [37], and even increase seroconversion rates in populations with reduced responsiveness [38]. Although widely used, their mechanisms of action are just now being uncovered. Still, few adjuvants have been approved for clinical use [39].

Aluminum salts (Alum) are by far the most widely used adjuvant since their introduction in the 1920s. However, for some vaccine formulations, they do not elicit protective and sustained immune responses. For that reason, substantial efforts have been devoted to the development of a new generation of compounds with stimulatory properties. Some of these new adjuvants are commercially available and already in advanced clinical trials. Among this new generation of adjuvants are classes of compounds recognized by different receptors present on cells of the innate immune system. The receptors they engage can be classified as: Toll-like receptors (TLR), NOD-like receptors (NLR), RIG-I-like receptors (RLR), and C-type lectin receptors (CLRs) [40]. Several TLRs agonists are in clinical development and expected to reach approval in the near future. Currently, the TLR4 agonist MPLA is licensed for human use in HPV vaccine. Moreover, CpG ODN 7909 (TLR9 agonist) are currently being used in an HBV vaccine formulation [41] while a poly (I:C) analog (TLR3 agonist) was evaluated as adjuvant for an intranasal H5N1 Influenza virus vaccine [42]. The agonists of TLR7/8 imiquimod (R837) and resiquimod (R848) have been tested in phase I using influenza and cancer antigens [43]. In addition, imiquimod is already approved for the topical treatment of genital warts, skin cancer, superficial basal cell carcinoma, and actinic keratosis.

Previous studies indicated that the use of different adjuvant formulations could dramatically influence the humoral response generated in response to HIV env antigens. For example, administration of the gp120 protein formulated with alum elicited a Th2 response characterized by high IgG1 production while the same vaccine antigen, administered together with the cytokine IL-12 induced a Th1 response with the production of IgG2 and IgG3 antibody isotypes [44]. The inclusion of adjuvants in a vaccine formulation can also lead to the generation of antibodies with improved functional attributes. The use of granulocyte-macrophage-colony stimulating factor (GM-CSF) as an adjuvant was able to induce higher avidity antibodies against env [45].

In an effort to better understand how adjuvants influence specific humoral and cellular immunity against env, we comprehensively compared the immunogenicity of the gp140 trimer subunit HIV vaccine with several adjuvant formulations. We tried to establish a hierarchy of the potency of adjuvants when admixed with the recombinant trimeric gp140. For that purpose, we compared the following TLR agonists: poly (I:C) (TLR3), MPL (TLR4), CpG ODN 1826 (TLR9), R837 (TLR7), and R848 (TLR7/8). In addition, we also used the NLR agonist MDP, aluminium hydroxide (Alum) and the gold standard Complete/Incomplete Freund’s adjuvant. Also, we assessed whether the combination of adjuvants has any additive effects on the magnitude or quality of humoral response. Lastly, we compared the levels of anti-gp140 antibodies induced by priming with a DNA vaccine encoding gp140 and boosting with gp140 in the presence of the adjuvant poly (I:C).

Herein, we show that vaccination with trimeric gp140 in the presence of different adjuvants can induce high-affinity antibody titers, follicular helper T cells and germinal center B cells. Taken together, the data show that poly (I:C) is the most potent adjuvant for priming Th1 immunity as evidenced by CMI responses. Furthermore, we demonstrate that combination of some adjuvants may exert additive effects that impact on the magnitude and quality of humoral responses while other combinations had no impact on total IgG titers but highly impact IgG subclass. In addition, heterologous DNA prime- protein boost in combination with poly (I:C) impact the magnitude of total IgG titers when compare to DNA alone and the quality of humoral response when compare to protein immunization as evidenced by IgG1/IgG2a ratio.

## Materials and Methods

### Protein expression and purification

The envelope gp140 glycoprotein used in this study was derived from the YU2 primary R5 clade B HIV-1 isolate. The expressing vector (pcDNA3.1- YU2) has been described in detail elsewhere [29] and was kindly provided by Dr. Michel C. Nussenzweig (The Rockefeller University, USA). The construct consists of the complete gp120 and gp41 ectodomains with mutations in the gp120/gp41 proteolytic cleavage site and a heterologous trimerization motif from T4 phage fibritin.

Recombinant trimeric gp140 was produced in HEK293T cells grown in Dulbecco’s modified Eagle’s medium (Invitrogen) supplemented with 1% (v/v) antibiotic-antimycotic (Invitrogen), 1% (v/v) l-glutamine (Invitrogen), and 5% (v/v) Ultra low IgG Fetal Bovine Serum (Invitrogen) that were transiently transfected with the plasmid pcDNA3.1- YU2 (20 μg) using polyethylenimine (PEI)-precipitation method (Sigma). After 4 days in culture at 37°C and 5% CO_2_, the culture supernatants containing secreted proteins were collected by centrifugation at 5,000 rpm for 20 minutes at 4°C, filtered through 22 μM membrane, supplemented with 100 μM phenylmethylsulfonyl fluoride (PMSF) and added to a dialysis membrane (Thermo Scientific). The protein was concentrated by the addition of sucrose until the solution volume was reduced to half. The gp140 trimers were purified by affinity chromatography using a cobalt (Co^++^) column (Thermo Scientific), dialyzed against PBS, resolved on a SDS-10% polyacrylamide gel, quantified and stored at -20°C until use.

### Immunoblot

Following SDS-PAGE, western blotting was performed. Trimer was detected using serum from a HIV-infected individual (1:2500), MAb (10–188) (1:10,000) [46] and sera from immunized mice (1:1000) followed by peroxidase labeled goat anti-mouse IgG (1:5000) or anti-human (1:2000) (Jackson Laboratories). The reaction was developed with TMB (Kirkegaard & Perry Laboratories).

### Immunization with the recombinant Env HIV-1 gp140 trimer

Six to eight week old female BALB/c (H-2d) mice were purchased from Centro de Desenvolvimento de Modelos Experimentais para Medicina e Biologia (CEDEME), Brazil. Mice were housed and manipulated under specific pathogen-free conditions in the animal care facilities of the Division of Immunology of Federal University of São Paulo. All procedures followed the National Institutes of Health Guide for the Care and Use of Laboratory Animals and were approved by the Institutional Animal Care and Use Committee (IACUC protocol number 0421/11) in accordance with the Brazilian National Law (11.794/2008). BALB/c mice were immunized twice, 2 weeks apart, with 10 or 1 μg of the recombinant protein in the presence of the indicated adjuvant formulations. Mice were injected subcutaneously (s.c.) in the 2 hind footpads with the recombinant antigen emulsified or mixed with adjuvant. A volume of 50 μL was injected into each hind footpad. Two weeks later, each animal received a booster injection of the same antigen s.c. at the base of the tail. None of the mice became severely ill or died prior to the experimental endpoint.

### Adjuvants

The trimer gp140 was diluted in saline and emulsified/mixed with the following adjuvants: i) 1 mg Monophosphoryl Lipid A (InvivoGen), ii) Complete/ Incomplete Freund’s adjuvant (CFA/IFA, Sigma) according to manufacturer specifications, iii) 50 μg polyinosine-polycytidyl IC- poly (I:C) (InvivoGen), iv) 20 μg Imiquimod- R837 (InvivoGen), v) 20 μg Resiquimod- R848 (InvivoGen), vi) 10 μg Muramyl dipeptide (MDP)- (InvivoGen), vii) 1mg aluminiun hydroxide gel (Sigma), viii) 20 μg Ribi (Sigma) and ix) 10 μg CpG ODN 1826 (TCCATGACGTTCCTGACGTT) synthesized with a nuclease-resistant phosphorothioate backbone (InvivoGen). In animals that received the first dose with Complete Freund’s adjuvant (CFA, Sigma), the booster injections were provided with antigen emulsified in IFA. The control groups received the adjuvant alone or the recombinant protein diluted in PBS.

### DNA vaccine and immunization

Plasmid encoding env protein (pcDNA3.1- YU2) was amplified using DH5α cells and isolated using the EndoFree Plasmid Giga Kit (Qiagen) according to manufacturer’s instructions. BALB/c mice were immunized intramuscularly (i.m) three times, two weeks apart, with 100 μg of the DNA vaccine diluted in saline (50 μL in each quadriceps). For heterologous prime- boost regimen, mice received one or two doses of the DNA vaccine followed by one or two doses of 10μg of gp140 in the presence of 50μg poly (I:C).

### Measurement of specific anti-gp140 antibodies in mouse serum

To determine anti-gp140 antibody titers in the sera, 250 ng of the YU2 gp140 glycoprotein diluted in carbonate/bicarbonate buffer were adsorbed onto the well of an enzyme-linked immunosorbent assay (ELISA) plate (high binding- Costar) overnight at 4°C. After washes with PBS-Tween (PBST) (0,02% v/v), wells were blocked with PBST, BSA (1% w/v) and non-fat milk (5% w/v), for 2 hours at rt. After this period, 100 μL of serially diluted serum from mice immunized with the envelope glycoprotein in the presence of different adjuvant formulations were applied to each well for 2 h at rt. Plates were washed 3 times with PBST. After 1-hour incubation with horseradish peroxidase (HRP) anti-mouse immunoglobulin G (IgG) (Jackson Laboratories), the plates were vigorously washed and the enzymatic reaction was developed by the addition of 1 mg/ml of o-phenylenediamine (Sigma) diluted in phosphate–citrate buffer, pH 5.0, containing 0.03% (v/v) hydrogen peroxide. The enzymatic reaction was stopped by the addition of 50 μL of a solution containing 4N H_2_SO_4_. Plates were read at 492 nm (OD_492_) with an ELISA reader (Labsystems Multiskan MS). The individual titers were considered as the highest dilution of serum that presented an OD_492_ higher than 0.1. The results are presented as the mean of log antibody titers + SD of 6 animals per group.

ELISA to detect the subclasses of mouse IgG was performed as described above, except that the secondary antibodies were antibodies specific for mouse IgG1, IgG2a and IgG2b (SouthernBiotech) diluted 1:4000.

### Measurement of anti-gp140 antibodies affinity

The affinity of anti-gp140 antibodies were assessed by a thiocyanate elution-based ELISA [47]. The procedure was similar to that described for the standard ELISA with the inclusion of an extra step. After the plates were washed following incubation of the pooled serum dilutions, ammonium thiocyanate was added to the wells in concentrations ranging from 0 to 8M. The plates were allowed to stand for 15 min at rt before they were washed and the assay proceeded. The concentration of ammonium thiocyanate required to dissociate 50% of the bound antibody was determined. The percentage of binding was calculated as follows: OD_492_ in the presence of ammonium thiocyanate X 100 / OD_492_ in the absence of ammonium thiocyanate.

### Spleen cell and lymph nodes isolation for immune assays

Two weeks after the last immunization, mice were euthanized under general anesthesia with a mixture of ketamine 300mg/kg and xylazine 30mg/kg via i.p. and spleens/ draining lymph nodes were removed aseptically. Single cell suspensions were obtained after red blood cell lysis with ammonium chloride potassium (ACK) solution. Cells were then resuspended in R-10 (RPMI 1640 supplemented with 10% of fetal bovine serum (Gibco), 2 mM L-glutamine (Gibco), 10 mM Hepes (Gibco), 1mM sodium pyruvate (Gibco), 1% v/v non-essential aminoacids solution (Gibco), 40 μg/mL of Gentamicin, 20 μg/mL of Peflacin and 5x10^-5^M 2- mercaptoetanol (Gibco). The viability of cells was evaluated using 0.2% Trypan Blue exclusion dye to discriminate between live and dead cells. Cell concentration was estimated with the aid of a cell counter (Countess- Invitrogen) and adjusted in cell culture medium.

### Germinal center B cells and follicular helper T-cell phenotyping by flow cytometry

To quantify germinal center (GC) B cells and follicular helper T-cells (Tfh) we stained isolated draining lymph node cells with anti-CD4 pacific blue, anti-B220 PercP, anti-PD-1 PE, anti-CXCR5 biotin, anti-CD95 PE, anti-GL7 FITC and streptavidin APC (all from BD Biosciences). One million events in a live lymphocyte gate were acquired on a FACSCanto flow cytometer (BD Biosciences) and then analyzed using FlowJo software (version 10, Tree Star). Germinal center B cells were identified as CD4^-^B220^+^GL7^+^CD95^+^ and Tfh as CD4^+^B220^-^PD1^+^CXCR5^+^ population. S1 Fig shows the gating strategy used to identify each cell population.

### B cell ELISpot assay

The frequency of antigen-specific memory B cells was determined as previously described [48]. Briefly, the gp140 glycoprotein diluted in PBS (250 ng/well) was used to coat nitrocellulose plates overnight at rt. Antibody secreting cells (ASC) were detected using HRP goat anti-mouse IgG (Jackson Laboratories) and the reaction was developed by AEC colorimetric substrate. Number of spots per well was counted using an automated stereomicroscope (KS ELISpot, Zeiss).

### T Cell ELISpot assay

Splenocytes from immunized mice were assayed for their ability to secrete IFN-γ after *in vitro* stimulation with recombinant gp140 (5 μg/mL) using the ELISPOT assay. The ELISPOT assay was performed using mouse IFN-γ ELISPOT Ready-SET-Go! (eBiosciences) according to manufacturer’s instructions. Spots were counted using an automated stereomicroscope (KS ELISpot, Zeiss). The cutoff was 15 SFU per million splenocytes.

### CFSE-based proliferation assay

To monitor the expansion and proliferation of HIV-specific T cells, splenocytes from immunized mice were labeled with carboxyfluorescein succinimidyl ester (CFSE) as previously described [49]. Cells were cultured with medium only or recombinant gp140 (5 μg/mL). After 5 days, cells were stained with anti-CD3 PE, anti-CD4 PerCP and anti-CD8 APC monoclonal antibodies (BD Pharmingen). Samples were acquired on a FACSCanto flow cytometer (BD Biosciences) and then analyzed using FlowJo software (version 10, Tree Star).

### Data analysis

The One-way ANOVA followed by the Tukey’s honestly significantly different (HSD) were used to compare the possible differences between the mean values of different groups. Statistical analysis and graphical representation of data were performed using GraphPad Prism version 5.0 software.

## Results

### Immunization with HIV-1 gp140 trimer in different adjuvant formulations elicit robust IgG responses

The recombinant trimeric form of gp140 was produced and after purification by affinity chromatography only the band with the expected molecular weight was detected (S2A Fig). To evaluate if the recombinant protein retained its antigenic properties, we performed an immunoblot using serum from a HIV-infected individual and a monoclonal antibody derived from memory B cells from individuals infected with clade A and B HIV virus that recognizes only the trimeric form of the gp140. We found that both serum from HIV-1 infected individual and the monoclonal antibody recognized only one band with the expected molecular weight showing that the pure recombinant protein retained its antigenic properties (S2B Fig). Next, to address the minimum number of doses to induce high antibody titers, groups of 6 mice were immunized with 1, 2 or 3 doses of 10 μg of the recombinant HIV-1 gp140 trimer in the presence of different adjuvants. We initially tested the TLR3 agonist poly (I:C), the TLR9 agonist CpG ODN 1826 and, as a gold standard, Complete/Incomplete Freund’s adjuvant (CFA/IFA). ELISA assessed envelope-specific binding antibodies throughout the course of the immunization schedule. We observed that serum anti-gp140 IgG titers increased significantly after the second dose when compared to the first dose and did not increase after a third dose, indicating that they had reached maximum serum concentrations (S3 Fig). This experiment led us to conclude that two doses of the recombinant gp140 trimer are sufficient to induce high specific antibody titers.

**Fig 1:**
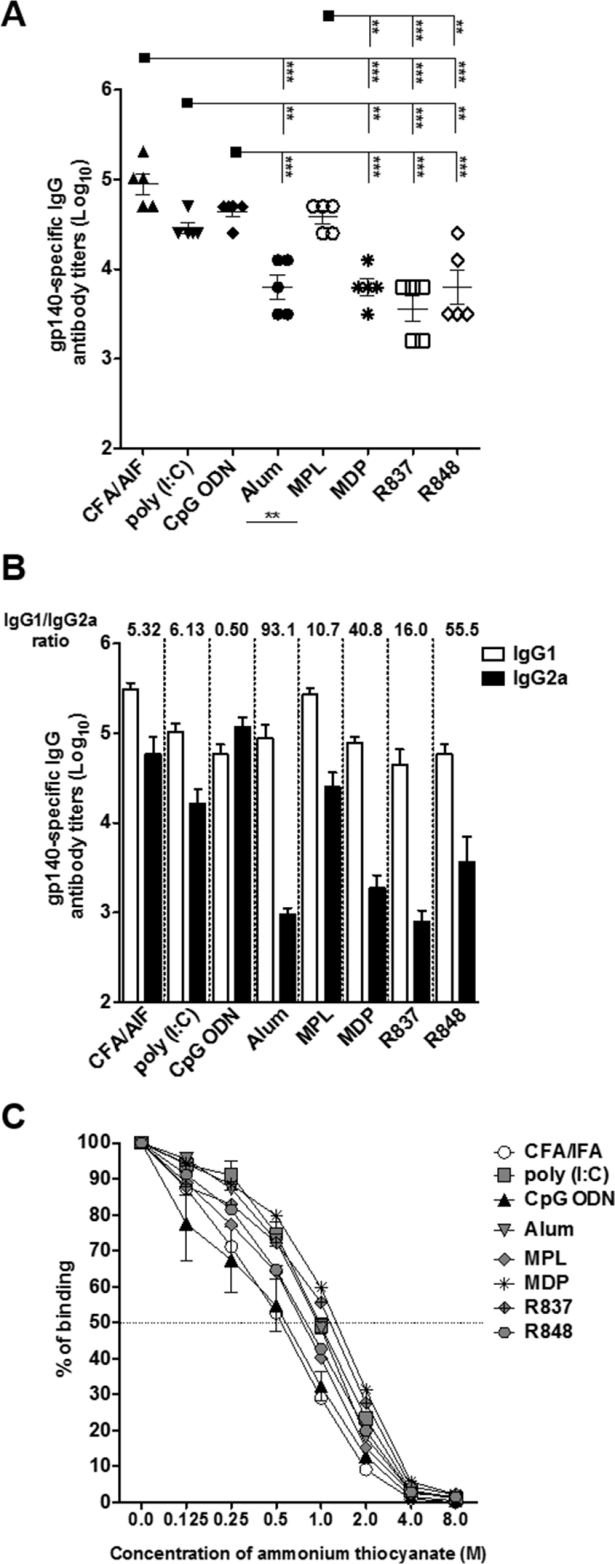
Immunization with Env HIV-1 gp140 trimer is able to elicit high-affinity specific antibody titers in the presence of different adjuvant formulations. BALB/c mice (n = 5 per group) received 2 doses with 10 μg of gp140 via s.c in the presence of the adjuvants CFA/IFA, poly (I:C), CpG ODN, alum, MPL, MDP, R837 or R848. Fifteen days after last dose, serum was collected and individually analyzed by ELISA. (A) Total gp140-specific IgG antibody titers on a logarithmic scale; (B) Specific IgG subtypes; (C) Antibody affinity was determined by ELISA with increasing concentrations of the chaotropic agent ammonium thiocyanate. **p< 0,01; *** p<0,001. Data represent mean ± SD.

We then tested whether other adjuvant formulations could modulate specific immune responses (Fig 1). For this purpose, mice were immunized twice with the gp140 trimer as established before in the presence of CFA/IFA, poly (I:C), CpG ODN, Alum, MPL, MDP, R837, and R848. We initially compared gp140-specific IgG titers (Fig 1A) and observed that immunization in the presence of CFA/IFA, poly (I:C), CpG ODN and MPL induced the highest antibody titers. There were no differences among these groups and all induced higher antibody titers when compared to Alum, MDP, R837 and R848. Control mice were immunized with adjuvant in diluent only or gp140 alone and did not present detectable levels of specific antibodies (S4 Fig). We next performed an immunoblot using pooled sera derived from all immunized mice (S5 Fig) and observed that antibodies present in the sera of mouse that received the antigen plus any of the adjuvants tested recognized the gp140 trimer. Analysis of specific IgG subtypes (Fig 1B) showed that CpG ODN immunized mice presented the lowest IgG1/IgG2a ratio (0.50) when compared to all other groups. It is of note that Alum, a typical Th2 adjuvant, induced the highest IgG1/IgG2a ratio.

To obtain an estimate of B cell maturation, the avidity of gp140-specific serum antibodies was measured using an ammonium thiocyanate ELISA (Fig 1C). Formulations with CpG ODN and CFA/IFA elicited antibody titers with the lowest avidities, as 0.48 and 0.50 M of ammonium thiocyanate were necessary to inhibit 50% of the binding, respectively. On the other hand, poly (I:C), Alum, MDP and R837 sera presented the highest avidities (0.94, 0.88, 1.14 and 0.94 M of ammonium thiocyanate, respectively, to inhibit 50% of the binding). For MPL and R848, 50% inhibition values were 0.66 and 0.73 M of ammonium thiocyanate, respectively. These results demonstrate that adjuvants play a critical role in the potency and quality of antibody responses after immunization with trimeric gp140.

We next tested whether adjuvants could modulate specific immune responses using ten times less antigen (1 μg of gp140). This dose represents approximately 4 μg of antigen per kg. We initially compared total gp140-specific IgG titers (S6A Fig) and observed that immunization with the adjuvant Ribi, that contains MPL and trehalose dicorynomycolate (TDM), induced the highest antibody titers even when compared to the gold standard CFA/IFA. There were no differences between CFA/IFA, poly (I:C) and CpG groups and all induced higher antibody titers when compared to alum, MDP, R837 and R848. All three specific IgG subtypes (IgG1, IgG2a, IgG2b) were detected in the tested groups (S6B Fig).

To investigate the longevity of humoral response after vaccination, we compared antibody titers in the sera from immunized mice up to 150 days after the administration of the second dose. Env-specific antibodies remained relatively constant in sera from mice immunized with gp140 trimer in all adjuvant formulations tested (S6C Fig). This result showed that immunization with the gp140 trimer in the presence of different adjuvants induced long-lived humoral responses.

### Gp140 formulated with several adjuvants induces follicular helper T cells, germinal center formation and memory B cells

**Fig 2:**
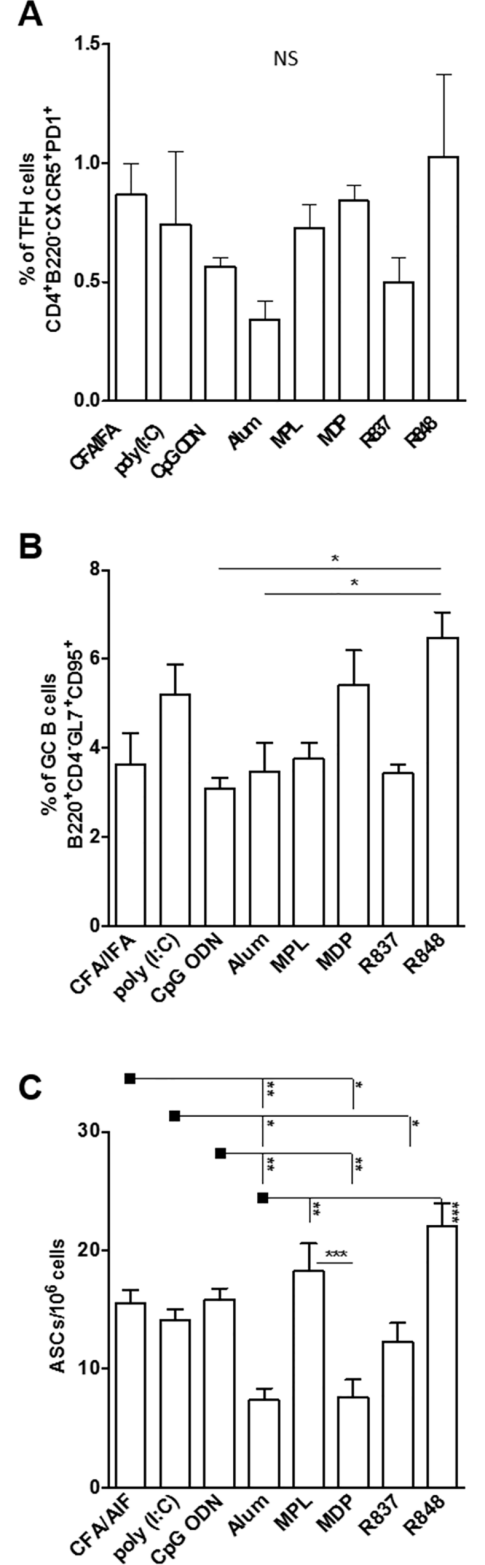
Immunization with Env HIV-1 gp140 trimer and adjuvants induces germinal center (GC) formation, follicular helper T cells (Tfh) and memory plasma cells. BALB/c mice (n = 5 per group) received 2 doses with 10 μg of gp140 via s.c in the presence of CFA/IFA, poly (I:C), CpG ODN, Alum, MPL, MDP, R837 or R848. Fifteen days after last dose, the draining lymph nodes were removed and Tfh and GC B cells were analyzed by flow cytometry, or cells were placed in culture for 16 hours to evaluate the number of gp140-specific antibody secreting cells by ELISpot. (A) Frequency of CD4^-^B220^+^CD95^-^GL7^+^ GC B cells; (B) Frequency of CD4^+^B220^-^PD1^+^CXCR5^+^ Tfh cells; (C) gp140-specific average spots per 1x10^6^ total cells as determined by ELISpot. *p< 0,05, **p<0,01; ***p<0,001, NS: non significant.

Follicular helper CD4^+^ T cells (Tfh) play a pivotal role in the antibody response because they deliver signals that drive the maturation and selection of B cells that generate increasingly potent antibodies. This process, known as affinity maturation, is critical to the generation of potent bNabs. Strong humoral responses, characterized by germinal center (GC) formation and memory B cells are highly dependent on help provided by Tfh [50]. We therefore analyzed whether immunization with the gp140 trimer and adjuvants affected the induction of Tfh cells, GC formation and frequency of memory B cells (Fig 2). We found Tfh cells by flow cytometry in the secondary lymphoid organs from mice immunized with all adjuvants tested (Fig 2A) with no statistical differences among the groups. Also, we detected GC B cells in lymph nodes from all immunized mice with statistical significance between R848 and Alum or CpG immunized groups (Fig 2B). In addition, a positive correlation was observed between total proportion of Tfh cells and GC B cells (data not shown). When we analyzed the presence of Tfh and GC B cells in lymph nodes from control mice immunized with adjuvant only or gp140 alone, we observed no more than 0.5% and 0.2% of cells, respectively (S7 Fig).

Memory B cells do not produce Abs unless they are reactivated by antigen or polyclonal stimulation to differentiate into antibody secreting cells (ASCs). To test if adjuvants can modulate this process we quantified the number of ASCs by enzyme-linked immunosorbent spot assay (ELISpot) using draining lymph node cells 15 days after the last immunization. As observed for total IgG titers, immunization with the gp140 trimer in the presence of CFA/IFA, poly (I:C), CpG ODN, and MPL presented the highest number of gp140-specific IgG secreting plasma cells when compared to all other groups (Fig 2C). Curiously, R848 was the only exception: while it presented high frequencies of Tfh, GC B cells and ASCs, the total IgG antibody titer from this group was statistically lower than the titers observed in the CFA/IFA, poly (I:C), CpG ODN, and MPL immunized groups (Fig 1A).

### Poly (I:C) is the most potent adjuvant for IFN-γ production and T cell proliferation

**Fig 3:**
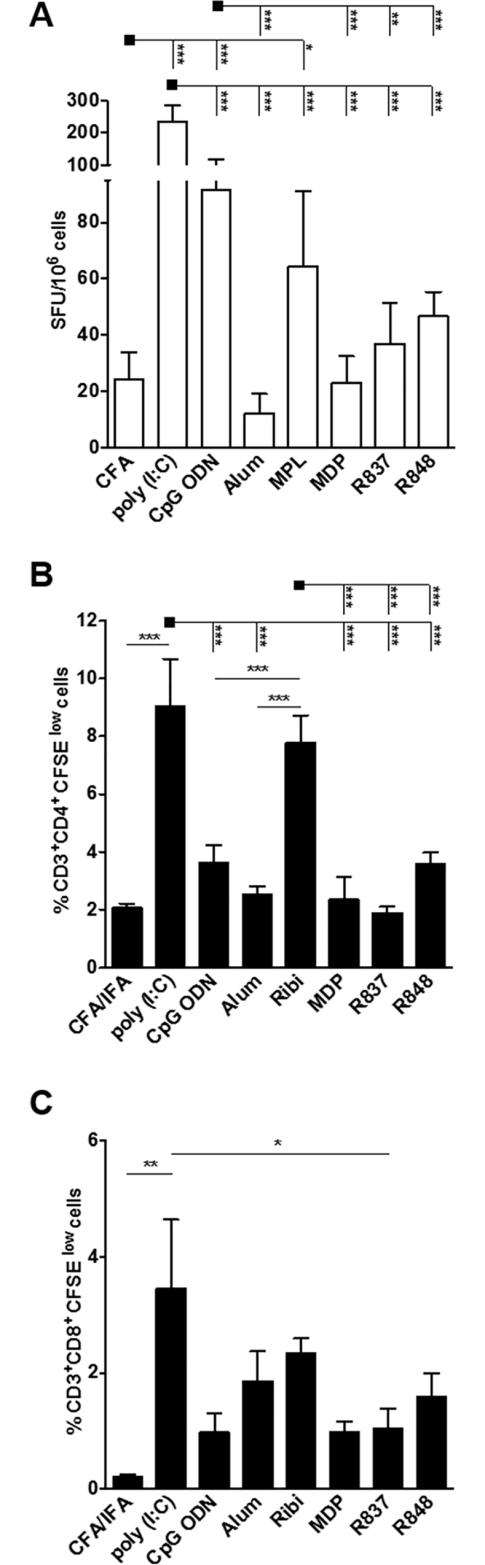
Immunization with Env HIV-1 gp140 trimer induces specific cellular mediated immune responses. BALB/c mice (n = 5 per group) received 2 doses with 10 or 1μg of gp140 via s.c in the presence of adjuvants. Fifteen days after the second dose, the spleen of each animal was removed and the splenocytes (A) were cultured in the presence recombinant protein gp140 (5 μg/mL) for 18 hours to evaluate the number IFN-γ producing cells by ELISpot assay, SFU: spot forming units; or were labeled with CFSE (B and C) and cultured in the presence of gp140 (5 μg/mL) for 5 days to evaluate specific proliferation. After staining with fluorochrome-labeled anti-CD3, -CD4 and -CD8 monoclonal antibodies, cells were analyzed by flow cytometry. CFSE dilution on gated (B) CD3^+^CD4^+^ or (C) CD3^+^CD8^+^ cells was used as readout for antigen-specific proliferation. Five hundred thousand events were acquired in a live lymphocyte gate. The percent of proliferating CD4^+^ and CD8^+^ CFSE^low^ cells was determined in the CD3^+^ cell population. The percentage of proliferating T cells was calculated subtracting the values of stimulated from non-stimulated cultures. *p< 0,05, **p<0,01; ***p<0,001. Data represent mean ± SD.

Cell- mediated immune response (CMI) is also a desirable feature as a component for an effective anti-HIV vaccine. To investigate whether immunization with gp140 trimer was able to induce specific T cell responses, we measured *in vitro* proliferation and the number of IFN-γ secreting cells 15 days after last immunization dose (Fig 3). Upon vaccination, gp140-specific IFN-γ secreting cells were detected in splenocytes from mice immunized with all adjuvants tested (Fig 3A), but with higher magnitude in splenocytes derived from poly (I:C), CpG ODN and MPL immunized mice. Of note, statistical difference was seen among the poly (I:C) immunized group and all the other groups, indicating that this adjuvant is particularly good at inducing IFN-γ producing cells.

We next sought to evaluate specific CD4^+^ and CD8^+^ T cell proliferation as also a measure of CMI responses. Analysis of proliferation revealed that 9% and 8% of CD4^+^ T cells from poly (I:C) and Ribi immunized groups proliferated, respectively, after stimulation with recombinant gp140 trimer (Fig 3B). CD8^+^ T cells also proliferated, albeit with lower magnitude. Fig 3C shows that poly (I:C) immunized mice presented a higher percentage of proliferating CD8^+^ T cells when compared to CFA/IFA and R837, while no difference was observed among all other groups. As observed for IFN-γ producing cells, poly (I:C) seems to be a suitable adjuvant to induce gp140 specific CD4^+^ and CD8^+^ T cell responses.

### Combination of adjuvants impacts the magnitude and the quality of Env-specific antibody responses

**Fig 4:**
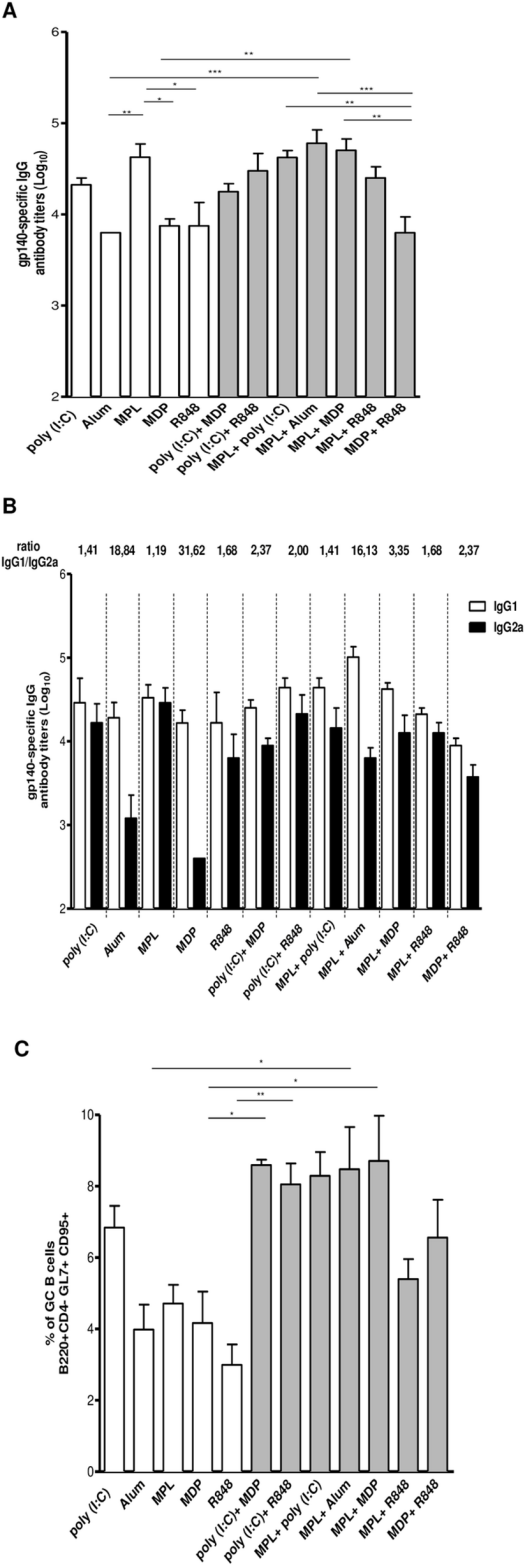
Combination of adjuvants exerts an additive effect on the immunogenicity of Env HIV-1 gp140 trimer. BALB/c mice (n = 5 per group) received 2 doses with 10 μg of gp140 via s.c formulated with adjuvants individually or in combination. Fifteen days after last dose, serum was collected and analyzed by ELISA. (A) Total gp140-specific IgG antibody titers on a logarithmic scale; (B) Specific IgG subtypes; (C) Fifteen days after last dose, the draining lymph nodes were removed and GC B cells were analyzed by flow cytometry.*p< 0,05, **p<0,01; ***p<0,001. Data represent mean ± SD.

To assess whether a combined adjuvant formulation would have any additive effect on the humoral response elicited by the recombinant protein, BALB/c mice were immunized with the gp140 trimer formulated with combinations of adjuvants. Two weeks after the last dose, we determined specific total IgG titers as well as IgG subclasses (Fig 4). Among all adjuvanted groups, only those that received the combination with MPL plus Alum and MPL plus MDP presented greater IgG antibody titers when compared to Alum or MDP vaccination alone (Fig 4A). Also, addition of MPL in Alum formulation or MPL plus MDP impacted on IgG subclasses as evidenced by IgG1/IgG2a ratio. Meanwhile, mixing MDP with poly (I:C) or with R848 had no impact on IgG titers but highly impacted on IgG subclass titers (from an IgG1/IgG2a ratio of 31.62 (MDP alone) to 3.35 and 2.37 respectively). This results demonstrate that some combination of adjuvants influence the total magnitude of IgG responses and/or alter class switching. On the other hand, we did not detect appreciable change when poly (I:C) was mixed with MPL or R848, showing that not all combinations had additive effects.

Analysis of GC B cells mirrored to some extent what we observed with antibody titers. When MPL was mixed with Alum the percentage of this cell population was higher when compared to Alum alone. The same pattern was observed with MPL plus MDP or MDP combined with poly (I:C) (Fig 4C). Taken together, these data support the conclusion that some adjuvant combinations have additive effect by inducing higher antibody titers and/or affect subclass pattern distribution by altering GC formation.

### Heterologous prime-boost alters the quality of Env-specific immune responses

**Fig 5:**
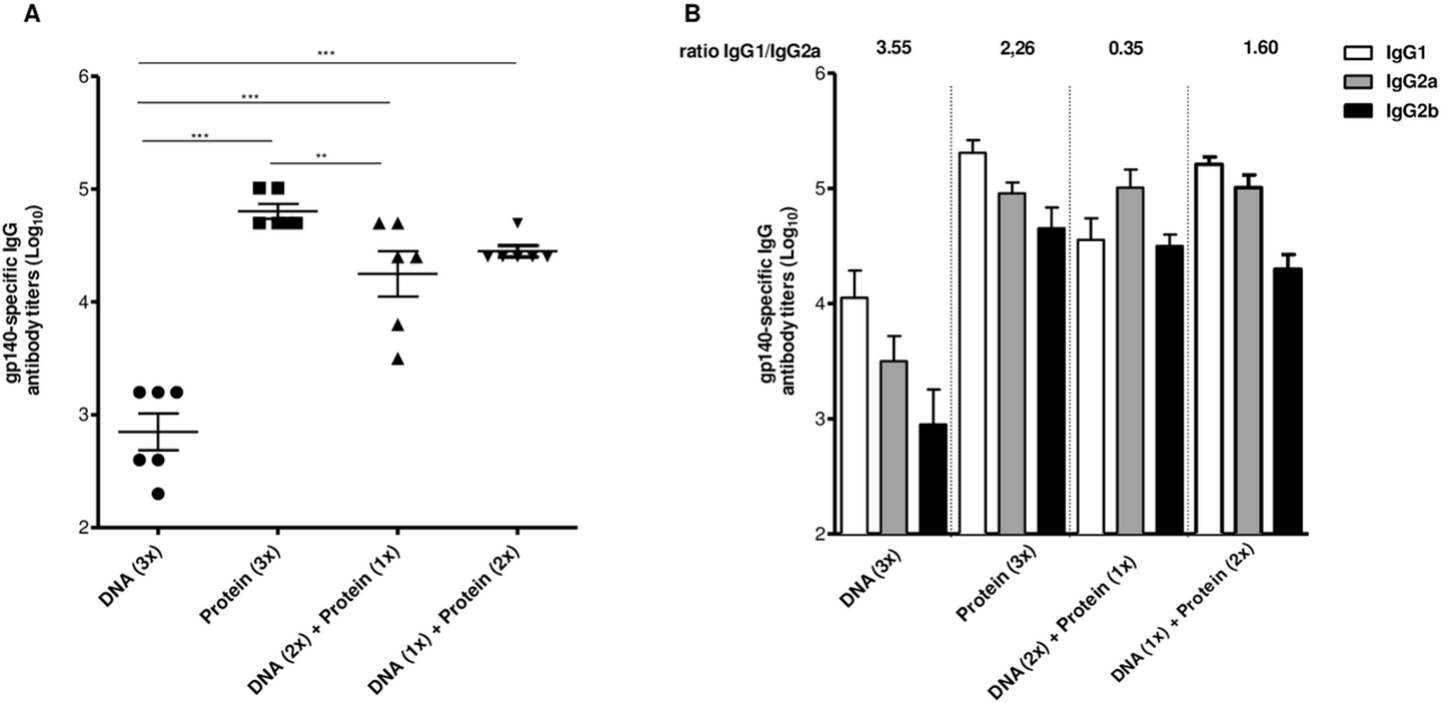
Heterologous prime-boost influences the quality of Env-specific humoral response. BALB/c mice (n = 6 per group) received 3 doses of the DNA vaccine (100 μg) encoding gp140 (Yu2-gp140) i.m. or 3 doses of gp 140 (10 μg) in the presence of poly (I:C) s.c. or 2 doses of DNA plus 1 dose of gp140 or 1 dose of DNA plus 2 doses of gp140. Fifteen days after last dose, serum was collected and analyzed by ELISA. (A) Total gp140-specific IgG antibody titers on a logarithmic scale; (B) Specific IgG subtypes. ***p<0,001.

Beside adjuvants, heterologous DNA prime-protein boost regimen has been used to enhance vaccine immunogenicity and/or efficacy. To test this hypothesis, we immunized mice with homologous or heterologous prime-boost using both DNA vaccine and the gp140 trimer in the presence of poly (I:C) (Fig 5). Antibody titers were enhanced by approximately 2 Logs with the inclusion of protein boost (one or two doses) after DNA prime when compared to homologous DNA immunization (Fig 5A). In addition, we did not observe difference among groups that received DNA prime-gp140 boost with homologous gp140 in combination with poly (I:C).

We next evaluated the pattern of IgG subclasses and observed that inclusion of a protein boost (one or two doses) after DNA priming decreased IgG1/IgG2a ratio indicating a skewed Th1 response when compared to homologous DNA or gp140 immunization (Fig 5B). This effect was more pronounced when mice received two doses of DNA for prime and one gp140 plus poly (I:C) dose for boost (IgG1/IgG2a ratio = 0.35). This result illustrate that heterologous DNA prime- protein boost altered the magnitude and quality of humoral response when compared to homologous DNA immunization and the quality of response when compare to homologous protein regimen.

## Discussion

An ideal vaccine against HIV should induce a humoral response comprising of Nabs, non-neutralizing antibodies and CMI. Such concept derived from studies performed with vaccinated individuals [18] [51] [24], vaccination/challenge experiments in non-human primates [52, 53], and long-term nonprogressor (LTNP) patients [54, 55]. In the present study we evaluate the impact of different adjuvant formulations on the immunogenicity of the HIV-1 gp140 trimer. The gp140 trimer produced in this study was also used to isolate B cells capable to produce Nabs from infected individuals that can control viremia and do not progress to AIDS [56] [46] [57].

Early attempts to induce an effective humoral response against HIV using env monomeric glycoprotein (Env gp120) administered with aluminium-hydroxide (Alum) (VAX04) were inefficient [14, 15] [16]. Several reports have previously demonstrated that Env gp140 trimers are able to elicit greater Nabs responses than Env gp120 monomers [58, 59]. Also, Env gp140 trimer induced potent cross-clade responses, a desired feature of a HIV-1 vaccine candidate [33]. HIV-1 recombinant Env protein-based vaccines are now primarily being used as a boost component in emerging HIV-1 vaccine strategies [17].

We demonstrated that immunization with Env gp140 trimer in the presence of several adjuvant formulations induced highly-specific antibody titers but CFA/IFA, poly (I:C), CpG ODN and MPL were superior to Alum, MDP, R837, and R848. By comparing the pattern of IgG subtypes, we observed that the same adjuvants (CFA/IFA, poly (I:C), CpG ODN and MPL) induced a more balanced Th1/Th2 response, while Alum induced the strongest Th2 type response.

Previous reports have evaluated the antibody responses to Env glycoproteins using different adjuvants [45, 60–65]. Immunization of mice with gp120 monomer in the presence CFA/IFA induced higher antibody titers than those observed after immunization with Ribi (MPL+TDM) [66]. Immunization of non-human primates with the gp140 trimer in the presence of Abisco 100 and CpG 2395 adjuvants was able to induce Nabs against viruses from clades A, B and C, and protected against SHIV challenge [67]. Most recently, a head-to-head comparison of multiple adjuvants was performed in an attempt to assess the stability and the immunogenicity of the clade C Env gp140 trimer. This study demonstrated that oil in water emulsions resulted in loss of structural integrity of the trimer, but regarding immunogenicity no single adjuvant exhibited superiority over others, although aluminum-based adjuvants induced lower responses [68].

The affinity of an antibody is highly influenced by B cell somatic hypermutation, and in HIV-infected individuals this phenomenon seems essential to increase antibody affinity and neutralization ability [69]. While under normal circumstances, high affinity of an antibody for its target is usually achieved after the accumulation 10–15 mutations, broad anti-HIV Nabs may contain 40–100 somatic mutations [70, 71]. When we evaluated the affinity of gp140-specific antibodies generated after immunization, we observed that formulation with CFA/IFA and CpG ODN elicited antibody titers with the lowest avidity. This result demonstrates that while CpG ODN and CFA/IFA were potent antibody inducers they failed to fulfill affinity criteria. On the other hand, poly (I:C), Alum, MDP and R837 induced antibodies with the highest avidities.

Another important feature of a vaccine formulation is its ability to induce long-lived immune responses. For example, immunity generated by the yellow fever vaccine is effective for ten years. Using ten times lower antigen, we detected high antibody titers in serum from immunized mice up to 150 days after the administration of the last dose.

We also took advantage of the mouse model to extend the evaluation of cellular immune responses in secondary lymphoid organs. We evaluated the presence of Tfh cells, as strong humoral immune responses characterized by GC formation and long-lived plasma cells are dependent on help provided by CD4^+^ Tfh cells [50]. Here we showed that immunization with Env gp140 mixed with adjuvants induces GCs and Tfh cells. Previous work demonstrated that the presence of Tfh in the lymph nodes of non-human primates correlates with the magnitude of the specific SIV IgG response, GC response and the avidity of SIV-specific IgG [72].

The quality of immune responses is a critical factor in defining protective responses. Analysis of gp140-specific T cell responses revealed that splenocytes from mice immunized with poly (I:C) presented the highest numbers of IFN-γ secreting cells. Preclinical studies in non-human primates showed that functionally relevant specific CD4^+^ and CD8^+^ T cell responses are an essential prerequisite for new vaccine candidates against HIV/SIV [53, 73–75]. In addition to these evidences in animal models, the RV144 clinical trial also showed that protected subjects presented CD4^+^ T cell responses against env [18, 24, 76]. In our study, we found that immunization with gp140 in the presence of poly (I:C) induced the highest CD4^+^ T cell proliferation rates and, to a lesser extent, CD8^+^ T cells.

Taken together, our results showed that poly (I:C) emerged as one of the best adjuvants among all the ones tested. It was among the adjuvants that induced the highest anti-gp140 IgG titers, induced a balanced Th1/Th2 response and good avidity. It was also able to induce Tfh cells and GC formation. When the number of ASCs was analyzed, poly (I:C) was only inferior to R848. CMI analysis showed that the highest number of IFN-γ secreting cells were detected in mice immunized with poly (I:C). The same was also true for CD4^+^ T cell proliferation. In mice, when a chimeric antibody containing the gag protein was administered in the presence of poly I:C, protective immune response to a vaccinia virus expressing gag was obtained [77]. Moreover, the strong effect of poly (I:C) on gp140 antibody titers is supported by previous study [64]. In rhesus macaques, poly (I:C) showed better potency as an adjuvant for responses to a protein antigen when compared to other adjuvants [78, 79].

Adjuvants may be combined to achieve a stronger effect or a more potent skewing of immune responses. Alum induces a strong Th2 response, but is rather ineffective against pathogens that require Th1–cell-mediated immunity. Currently, much effort is devoted to combine alum with TLR9 agonists [80]. Additionally there is substantial interest in combining TLR agonists as vaccine adjuvants to enhance immunogenicity and efficacy. In experimental models, administration of combinations such as CpG ODNs with MDP or MPL has proven effective [81].

We combined several adjuvants and observed that combining MPL with Alum elicited greater antibody titers when compared to Alum vaccination alone, and this was accomplished by a change in IgG subtypes. Also, addition of MPL in MDP formulation impacted the magnitude of specific antibody titers with an expressive effect on IgG subclass as evidenced by the change in IgG1/IgG2a ratio. Meanwhile, mixing MDP with poly (I:C) or R848 had no impact on IgG titers but highly impacted IgG subclass titers. We also observed that not all combinations had additive effects. Of note, this knowledge can be applied to skew the immune response to a more effective profile.

Heterologous prime-boost has been extensively studied as a vaccination regimen to increase the magnitude and quality of the immune response against many pathogens, including HIV [82, 83]. Previous work in rabbits, demonstrate that a DNA prime- gp120 boost yielded antibodies with higher avidity and specificity and with improved neutralizing characteristics than homologous regimen [84–86]. An example of a prime-boot protocol in the clinic is the ongoing phase II study that uses gp140 in combination with aluminum phosphate as a boost (www.clinicaltrials.gov, access number: NCT02315703). Still others prime-boost trials uses gp140 in combination with the adjuvant MF59 or glucopyranosyl lipid A (GLA) (www.clinicaltrials.gov, access numbers: NCT01418235, NCT00073216, NCT01922284).

Using similar approach, we observed that heterologous DNA prime-gp140+ poly (I:C) boost exhibited superiority over homologous DNA immunization but not against homologous protein immunization regarding humoral responses. On the other hand, we observed that the quality of humoral response were improved when compare to protein immunization alone as evidenced by IgG1/IgG2a ratio.

When taken together our data show that the adjuvant poly (I:C) induced long-lived, high affinity antibody titers, Th1 skewed immune response, germinal center B cells, follicular helper T cells, CD4^+^ and CD8^+^ T cell responses. In summary, poly (I:C) provided the best humoral/cellular response combination and future experiments in non-human primates will mostly allow to extend such observations. The final fulfillment for an efficacious vaccine candidate will require continuous studies designed to address the best antigen/adjuvant combination to be used.

## Supporting Information

S1 Fig Representative flow cytometry dot plots for Tfh and GC populations based on CXCR5/ PD-1 and GL7/CD95 expression respectively. (TIF)

S2 Fig Analysis of recombinant trimer gp140 after purification by affinity chromatography. (A) SDS-10% polyacrylamide gel under reducing conditions of the recombinant gp140 trimer after purification using Cobalt column. Lane 1: flow through, lane 2: purified protein. (B) Immunoblot using serum from a HIV-infected patient and a monoclonal antibody.
(TIF)

S3 Fig Kinetics of antibody response to gp140. BALB/c mice (n = 4 per group) received 3 doses with 10μg of gp140 via s.c in the presence of the adjuvants CFA/IFA, poly (I:C) or CpG ODN. Fifteen days after each dose, serum was collected and individually analyzed by ELISA. Total gp140-specific IgG antibody titers on a logarithmic scale. *** p<0,001. Data represent mean ± SD.
(TIF)

S4 Fig Antibody titers in mice immunized with different adjuvant or gp140 alone. BALB/c mice (n = 5 per group) received 2 doses of the adjuvants CFA/IFA, poly (I:C), CpG ODN, alum, MPL, MDP, R837 or R848 or gp140 diluted in PBS. Fifteen days after last dose, serum was collected and individually analyzed by ELISA. Total gp140-specific IgG antibody titers on a logarithmic scale. Data represent mean ± SD.
(TIF)

S5 Fig Immunogenic properties of Env HIV-1 gp140 trimer in the presence of different adjuvant formulations. One microgram of recombinant gp140 trimer was resolved on a SDS-10% polyacrylamide gel and transferred to PVDF membrane for Immunoblot analysis. After blocking, the membrane was incubated with serum from BALB/c mice that received 2 doses with 10μg of gp140 via s.c in the presence of the adjuvants CFA/IFA, poly (I:C), CpG ODN, alum, Ribi, MDP, R837 or R848. Lane 1: adjuvant alone; lane 2: gp140 alone; lane 3: CFA/IFA + gp140; lane 4: Poly (I:C) + gp140; lane 5: CpG ODN + gp140; lane 6: alum + gp140; lane 7: Ribi + gp140; lane 8: R837 + gp140; lane 9: R848 + gp140.
(TIF)

S6 Fig Immunization with Env HIV-1 gp140 trimer is able to elicit high specific antibody titers in the presence of different adjuvant formulations with ten times less antigen. BALB/c mice (n = 8 per group) received 2 doses with 1μg of gp140 via s.c in the presence of the adjuvants CFA/IFA, poly (I:C), CpG ODN, alum, Ribi, MDP, R837 or R848. Fifteen days after last dose, serum was collected and analyzed by ELISA. (A) Total gp140-specific IgG antibody titers on a logarithmic scale; (B) Specific IgG subtypes; (C) Serum were collected until 150 days after last dose to analyze the longevity of humoral response by ELISA**p< 0,01; *** p<0,001. Data represent mean ± SD.
(TIF)

S7 Fig Frequency of Tfh and GC B cells in mice immunized with several adjuvants or gp140 alone. BALB/c mice (n = 5 per group) received 2 doses of the adjuvants CFA/IFA, poly (I:C), CpG ODN, Alum, MPL, MDP, R837 or R848 or gp140 diluted in PBS. Fifteen days after last dose, the draining lymph nodes were removed and Tfh and GC B cells were analyzed by flow cytometry. (A) Frequency of CD4^+^B220^-^PD1^+^CXCR5^+^ Tfh cells; (B) Frequency of CD4^-^B220^+^CD95^-^GL7^+^ GC B cells. NS: non significant.
(TIF)
