# Supplementary figures and images for: HIV Envelope Trimer Specific Immune Response Is Influenced by Different Adjuvant Formulations and Heterologous Prime-Boost
Source: PLoS One. 2016 Jan 4;11(1):e0145637. doi: 10.1371/journal.pone.0145637 (PMC4699765; doi:10.1371/journal.pone.0145637)

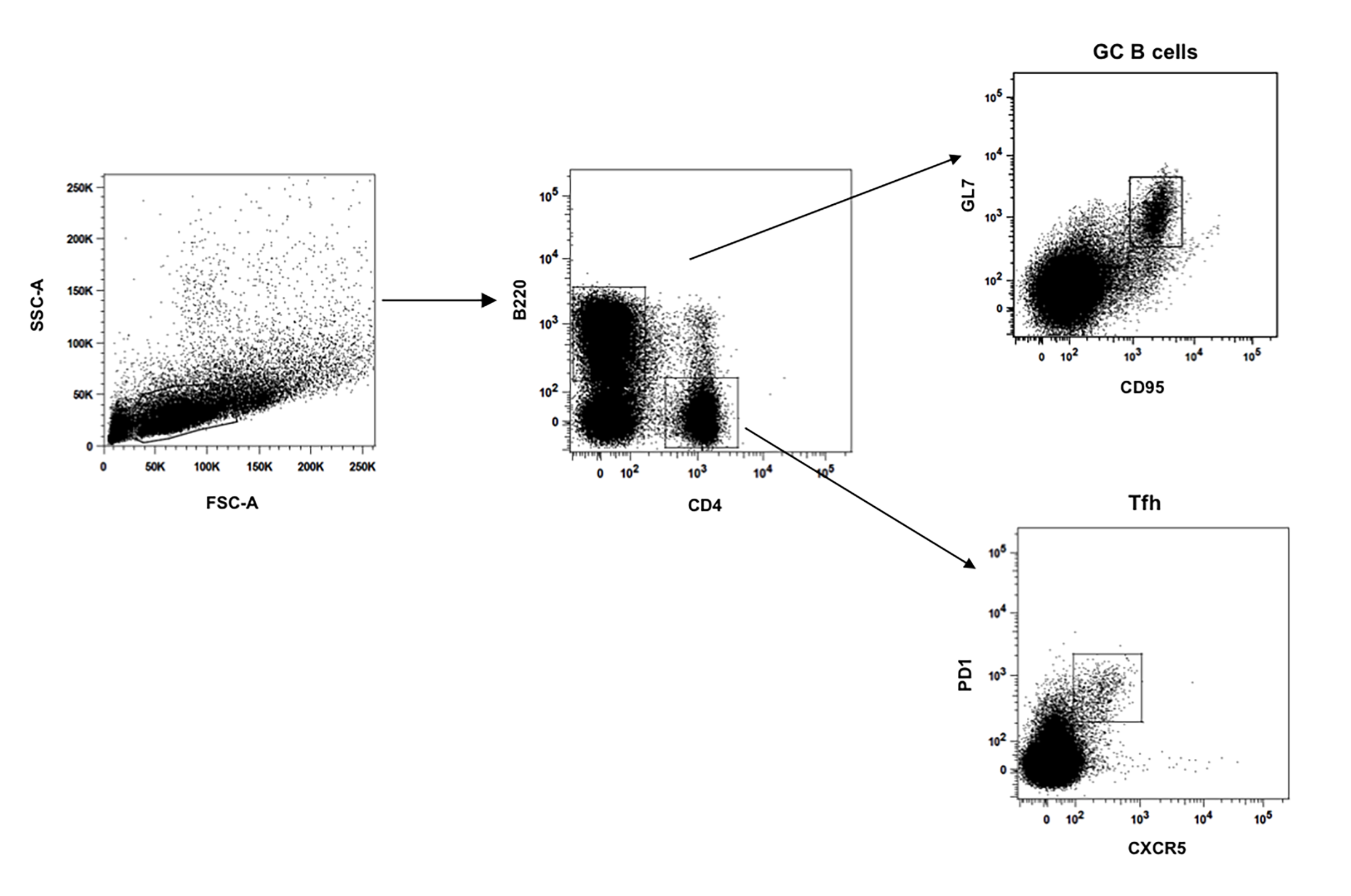

Supplement: S1 Fig — (TIF) [file pone.0145637.s001.tif]

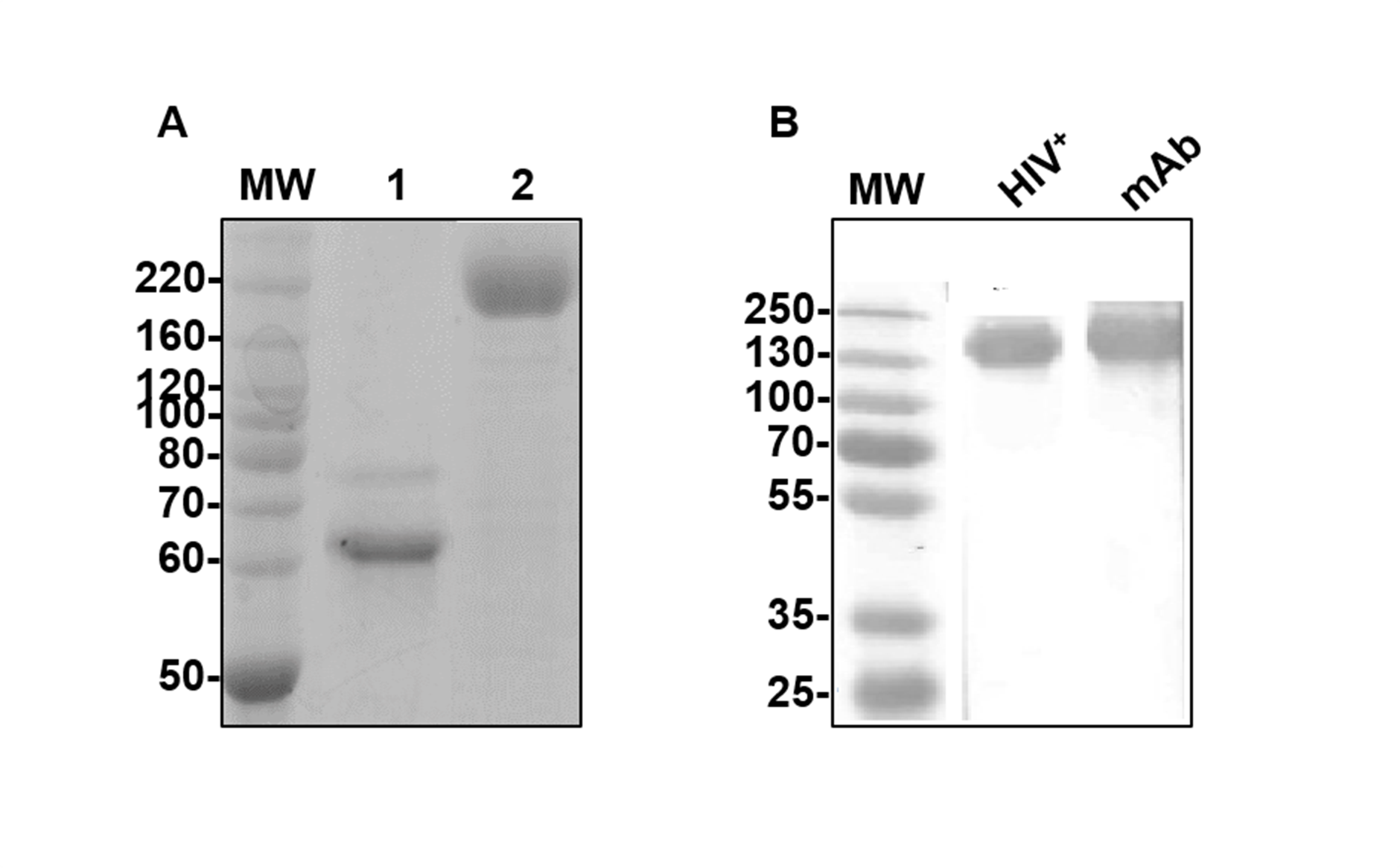

Supplement: S2 Fig — (A) SDS-10% polyacrylamide gel under reducing conditions of the recombinant gp140 trimer after purification using Cobalt column. Lane 1: flow through, lane 2: purified protein. (B) Immunoblot using serum from a HIV-infected patient and a monoclonal antibody. (TIF) [file pone.0145637.s002.tif]

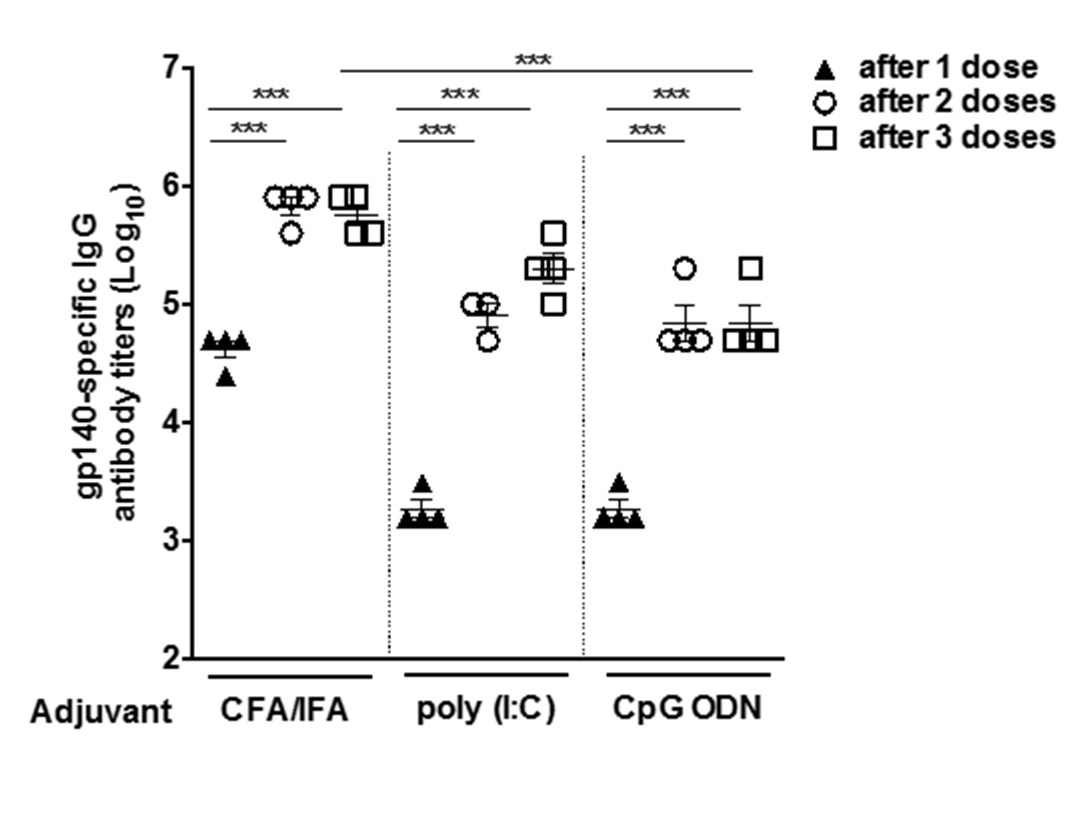

Supplement: S3 Fig — BALB/c mice (n = 4 per group) received 3 doses with 10μg of gp140 via s.c in the presence of the adjuvants CFA/IFA, poly (I:C) or CpG ODN. Fifteen days after each dose, serum was collected and individually analyzed by ELISA. Total gp140-specific IgG antibody titers on a logarithmic scale. *** p<0,001. Data represent mean ± SD. (TIF) [file pone.0145637.s003.tif]

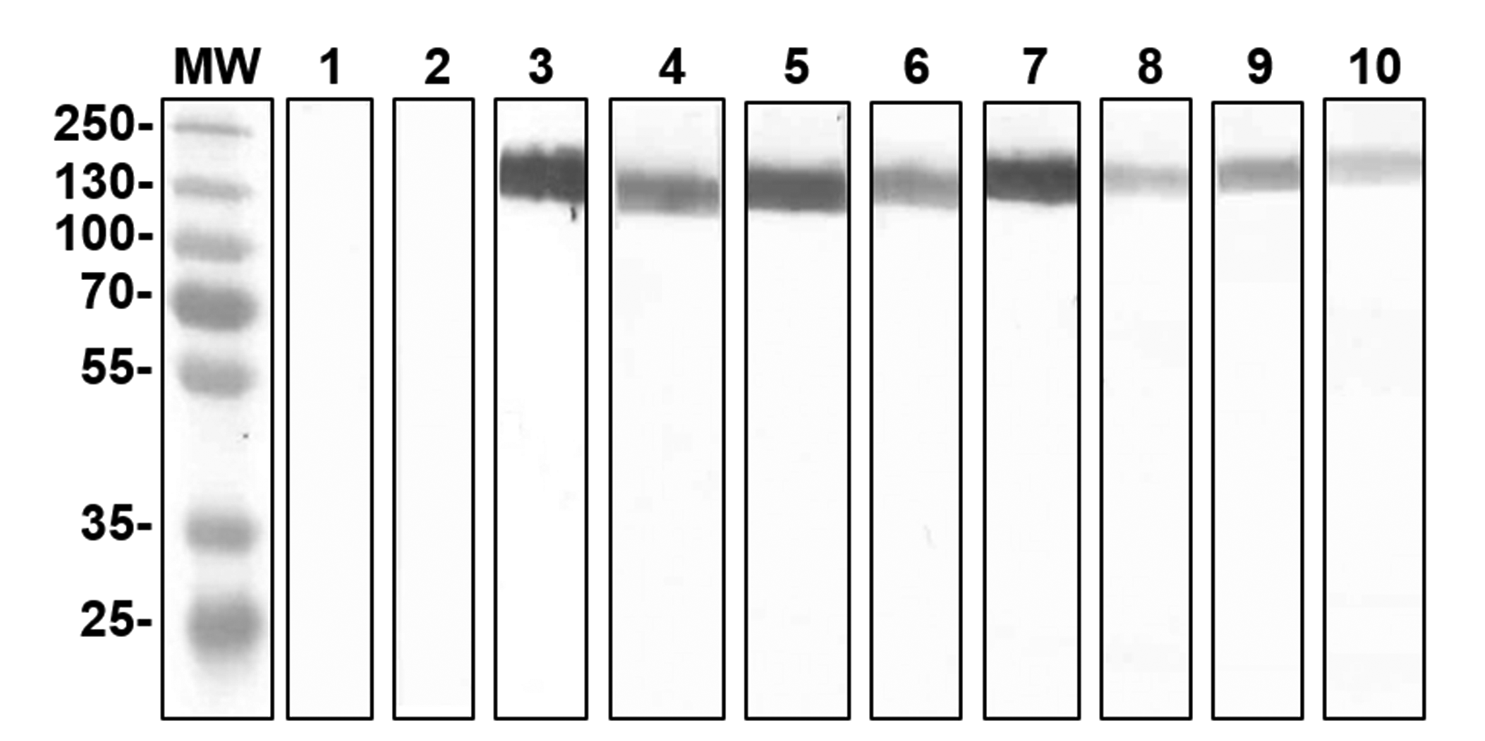

Supplement: S5 Fig — One microgram of recombinant gp140 trimer was resolved on a SDS-10% polyacrylamide gel and transferred to PVDF membrane for Immunoblot analysis. After blocking, the membrane was incubated with serum from BALB/c mice that received 2 doses with 10μg of gp140 via s.c in the presence of the adjuvants CFA/IFA, poly (I:C), CpG ODN, alum, Ribi, MDP, R837 or R848. Lane 1: adjuvant alone; lane 2: gp140 alone; lane 3: CFA/IFA + gp140; lane 4: Poly (I:C) + gp140; lane 5: CpG ODN + gp140; lane 6: alum + gp140; lane 7: Ribi + gp140; lane 8: R837 + gp140; lane 9: R848 + gp140. (TIF) [file pone.0145637.s005.tif]

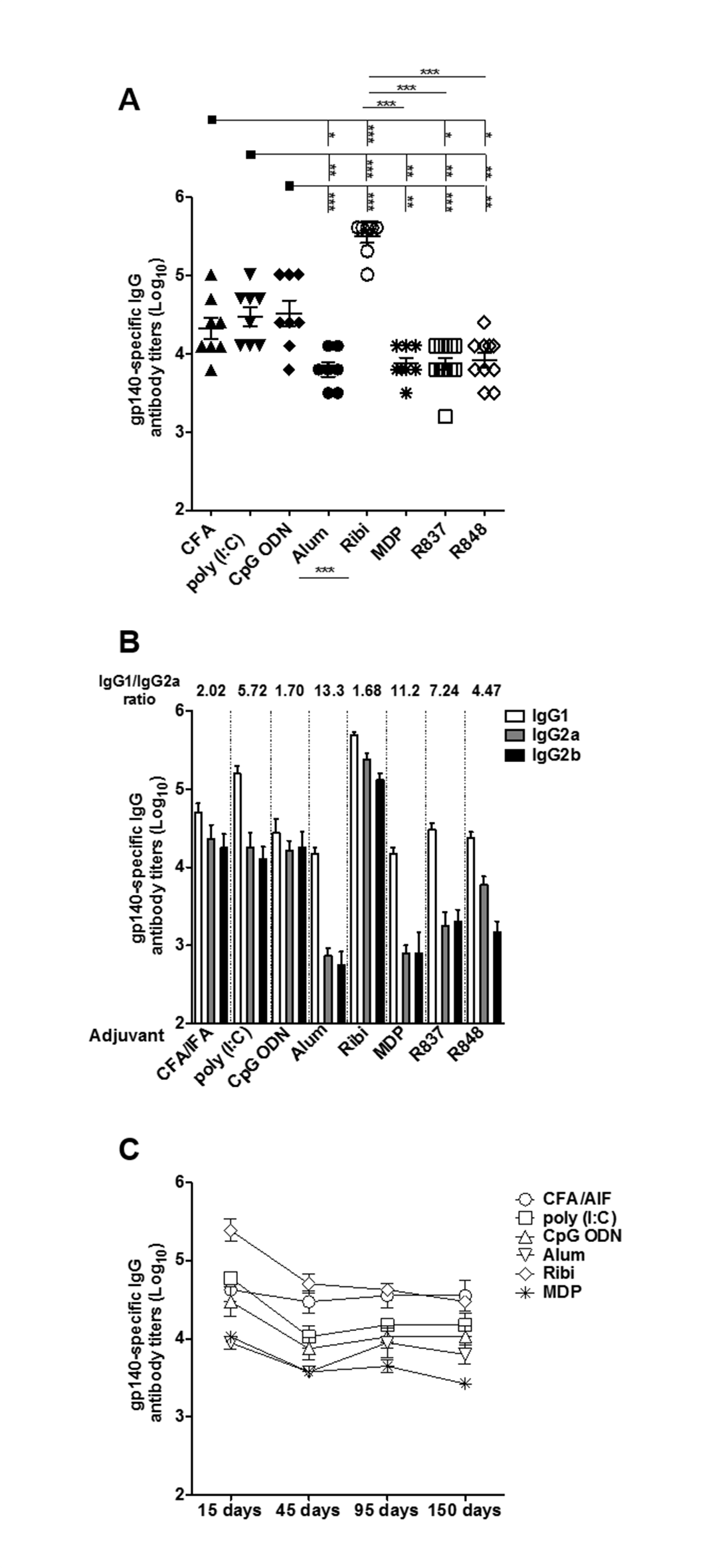

Supplement: S6 Fig — BALB/c mice (n = 8 per group) received 2 doses with 1μg of gp140 via s.c in the presence of the adjuvants CFA/IFA, poly (I:C), CpG ODN, alum, Ribi, MDP, R837 or R848. Fifteen days after last dose, serum was collected and analyzed by ELISA. (A) Total gp140-specific IgG antibody titers on a logarithmic scale; (B) Specific IgG subtypes; (C) Serum were collected until 150 days after last dose to analyze the longevity of humoral response by ELISA**p< 0,01; *** p<0,001. Data represent mean ± SD. (TIF) [file pone.0145637.s006.tif]
